# Autoantibodies Associated With Connective Tissue Diseases: What Meaning for Clinicians?

**DOI:** 10.3389/fimmu.2018.00541

**Published:** 2018-03-26

**Authors:** Kevin Didier, Loïs Bolko, Delphine Giusti, Segolene Toquet, Ailsa Robbins, Frank Antonicelli, Amelie Servettaz

**Affiliations:** ^1^Department of Internal Medicine, Infectious Diseases, and Clinical Immunology, Reims Teaching Hospitals, Robert Debré Hospital, Reims, France; ^2^Rheumatology Department, Maison Blanche Hospital, Reims University Hospitals, Reims, France; ^3^Laboratory of Dermatology, Faculty of Medicine, EA7319, University of Reims Champagne-Ardenne, Reims, France; ^4^Laboratory of Immunology, Reims University Hospital, University of Reims Champagne-Ardenne, Reims, France; ^5^Department of Internal Medicine, CHU de Reims, Reims, France; ^6^Department of Biological Sciences, Immunology, UFR Odontology, University of Reims Champagne-Ardenne, Reims, France

**Keywords:** antibody, systemic lupus erythematosus, Sjögren’s syndrome, systemic sclerosis, antisynthetase syndrome, dermatomyositis, necrotizing myopathy, rheumatoid arthritis

## Abstract

Connective tissue diseases (CTDs) such as systemic lupus erythematosus, systemic sclerosis, myositis, Sjögren’s syndrome, and rheumatoid arthritis are systemic diseases which are often associated with a challenge in diagnosis. Autoantibodies (AAbs) can be detected in these diseases and help clinicians in their diagnosis. Actually, pathophysiology of these diseases is associated with the presence of antinuclear antibodies. In the last decades, many new antibodies were discovered, but their implication in pathogenesis of CTDs remains unclear. Furthermore, the classification of these AAbs is nowadays misused, as their targets can be localized outside of the nuclear compartment. Interestingly, in most cases, each antibody is associated with a specific phenotype in CTDs and therefore help in better defining either the disease subtypes or diseases activity and outcome. Because of recent progresses in their detection and in the comprehension of their pathogenesis implication in CTD-associated antibodies, clinicians should pay attention to the presence of these different AAbs to improve patient’s management. In this review, we propose to focus on the different phenotypes and features associated with each autoantibody used in clinical practice in those CTDs.

## Introduction

Connective tissue diseases (CTDs) are autoimmune diseases characterized by the involvement of several organs and the presence of various autoantibodies (AAbs). Their implication in the pathogenesis of these CTD remains partly unclear; nevertheless, we know that some of these AAbs are directly involved in tissue damages whereas some are just markers of disease development.

During the last decades, many improvements were made in the comprehension of CTD pathogenesis, and a lot of new AAb were described. The presence of AAb can help the clinician in his approach to search an autoimmune disease (1), as sometimes the production of specific AAb precedes the symptoms and the diagnosis of the CTD (2, 3). Indeed, in most cases, those AAbs are detected in a specific CTD, making the diagnosis easier. Actually, most studies recently published focused on the clinical impact of AAb in different CTD and found that some AAbs are clearly associated with a specific phenotype in one type of CTD, allowing the clinician to adapt the follow-up of his patient and to predict some complications. However, relationship between AAb presence and disease diagnosis is not always that simple, as some other AAbs can be associated with more than one disease. Furthermore, differences can exist for the same kind of CTD according to the population studied, strengthening the fact that genetical factors in CTD pathogenesis are probably more important than we actually know. A potential explanation to these variations may be related to genetic and environmental factors, which may play a key role in these diseases predisposition and outcome.

Indeed, pathogenesis of CTD seems associated with the presence of AAb. However, many new AAbs were discovered, but their implication in pathogenesis of connective tissue diseases (CTDs) remains unclear. Many of these AAbs are antinuclear antibody (ANA). Nevertheless, the classification of ANA is nowadays misused, as their targets can be localized outside of the nuclear compartment (cytoplasmic, membrane, or extracellular), even if the term ANA is still currently used in clinic.

Because of the new improvements in their detection and comprehension of their pathological implication in CTDs-associated antibodies, clinicians should pay attention to the presence of the different AAbs to improve patient’s management. In this review, we propose to focus on the different phenotypes and features associated with each AAb used in clinical practice in CTD clearly defined such as systemic lupus erythematosus (SLE), Sjögren’s syndrome (SS), systemic sclerosis (SSc), myositis, and rheumatoid arthritis (RA). Especially, we will highlight the usefulness of their clinical determination.

## AAb in Healthy Population and in Non-Autoimmune Diseases

Biological autoimmunity is not always pathological and can be observed in healthy people. The highlighting of ANA in the general population is common and estimated between 5.92 and 30.8% (4–13) with a lower prevalence in the Chinese population (4) and a higher prevalence in the Afro-American population (13) (Table 1). In addition, ANAs are more commonly detected in women than in men (4–8, 10–14), and the prevalence of such ANA increases with aging, as it reaches up to 24% in subjects older than 85 years (14). ANAs are commonly detected by indirect immunofluorescence (IIF) on HEp2 cells, a human HELA-derivative cell line. Importantly, the relevance of a positive ANA test is directly linked to its titration. Thus, in a normal population, ANAs were found positive in 31.7% of individuals at 1/40 serum dilution, 13.3% at 1/80, 5.0% at 1/160, and 3.3% at 1/320 (15). The most accepted threshold is often the dilution 1/160 for first screening dilution (15–17). In complement to IIF assay, which is a very sensitive technic and can now be automated (18, 19), screening fluorescence enzyme or chemiluminescence immunoassays have been proposed in the last few years as detection assays. These multiparametric immunoassays allow simultaneous testing for 13–17 of commonest pathogenic autoantibody specificities in systemic autoimmune diseases [i.e., SSA-52kD, SSA-60kD, SSB, U1RNP (RNP 70,A,C), CENP-B, Scl70, Jo1, Fibrillarin, RNA polymerase III, ribosomal proteins, PM-Scl, PCNA, Mi2 proteins, Sm, dsDNA, and chromatin]. These screening immunoassays showed relatively good concordance with IIF (75–83%) and demonstrated similar or improved specificity and positive predictive value depending on the studies and the assays (20–24). However, due to the limited number of represented antigens in some screening assays and the better sensitivity of IIF, the American College of Rheumatology (ACR) ANA Task Force recommended that IIF should remain the gold standard for ANA testing (25).

**Table 1:** Presence of antinuclear antibody (ANA) in different populations considered as healthy people.

Reference	Population	Number	ANA positivity (%)	1/40Nb (%)	1/80Nb (%)	1/160Nb (%)	1/320Nb (%)	1/640Nb (%)	1/1,280Nb (%)	1/2,560Nb (%)
Wang et al. (4)	Chinese	20,970	5.92	886 (4.23)	105 (0.50)	77 (0.37)	55 (0.26)	29 (0.14)	36 (0.17)	53 (0.25)
Minz et al. (5)	Indian	36,310	12.3	–	–	–	–	–	–	–
Selmi et al. (6)	Italian	2,690	18.1	–	–	–	–	–	–	–
Fernandez et al. (7)	Brazilian	500	22.6	73 (14.6)	23 (4.6)	10 (2.0)	1 (0.2)	–	2 (0.4)	2 (0.4)
Peene et al. (8)	Belgian	10,550	23.5	–	–	–	–	–	–	–
Hayashi et al. (10)	Japanese	2,181	25.9	–	–	–	–	–	–	–
Racoubian et al. (11)	Lebanese	10,814	26.4	–	2,162 (20.0)	–	400 (3.7)	183 (1.7)	119 (1.1)	–
Roberts-Thomson et al. (12)	Australian	20,205	28.3	–	–	–	–	–	–	–
Wandstrat et al. (13)	Afro-American	1,827	30.8	–	–	–	–	–	–	–

*ANA positivity was defined as the first titer seen in this table for each study*.

In most healthy individuals with ANA, the antigenic target(s) remain(s) unknown with standard tests used to identify ANA subtypes. Nevertheless, in a minority of cases, AAbs from healthy people recognize the same autoantigens as AAb from patients with autoimmune disease, especially anti-SSa in up to 3% and anti-DFS70 AAb (also called LEDGF for “lens epithelium-derived growth factor”) (4, 10, 11). Anti-SSa AAbs are frequently detected in the sera from patients with SLE and SS, whereas anti-DFS70 AAbs have mostly been evidenced in healthy people, but also in the sera from patients with benign and common diseases such as atopic dermatitis (26–29). In general population, anti-Ro/SSa AAbs are associated with torsade de pointes (TdP) and arrhythmia, representing a clinically silent novel risk factor for TdP development *via* an autoimmune-mediated electrophysiological interference with the hERG channel (30).

Antinuclear antibody and other AAbs can also be observed in association with drugs (such as hydralazine and procainamide) or in non-autoimmune diseases associated with a process of tolerance breakdown such as infectious or lymphoproliferative diseases.

## Systemic Lupus Erythematosus-Associated AAb

Systemic lupus erythematosus is a CTD with a great variability in its clinical presentation and its prognosis. Two main classification criteria are available, based on the presence of both clinical and immunological parameters [1997 ACR classification criteria and Systemic Lupus International Collaborating Clinics (SLICC) classification criteria (31, 32)]. The different AAbs associated with SLE and their main features are recapitulated in Table 2.

**Table 2:** AAb associated with systemic lupus erythematosus (SLE).

AAb	Prevalence	Sensitivity	Specificity	Clinical features
Anti-dsDNA	43–92% (37–39)	8–54% (40–46)	89–99% (40–46)	Correlation with disease activity
Anti-nucleosome	59.8–61.9% (53–57)	52–61% (53–57)	87.5–95.7% (53–57)	Correlation with disease activity
Anti-Sm	15–55.5% (37–39, 61)	10–55% (62)	98–100% (62)	Most specific antibody in SLE often associated with anti-RNP AAb
Anti-histone	50–81%>90% in induced SLE (37, 71, 72)	–	–	Drug-induced SLE
Anti-C1q	4–60% (90–93)	28% (94–97)	92% (94–97)	Associated with glomerulonephritis
Anti-ribosomal P	12–60% (37, 103, 104)	36% (103, 104, 109)	97–100% (103, 104, 109)	Neuropsychiatric manifestations
Anti-Ro/SSa	36–64% (37, 38, 61, 75, 76)	–	–	Skin involvement+++CHB
Anti-La/SSb	8–33.6% (37, 38, 61, 75, 76)	25.7% (85)	96.7% (85)	Skin involvement+++CHB (less than anti-Ro AAb)
Anti-RNP	23.3–49% (37–39)	8–69% (62)	25–82% (62)	–

*CHB, congenital heart block; anti-dsDNA, anti-double-stranded DNA; AAb, autoantibody*.

### ANA in SLE

Antinuclear antibody is one of the immunological criteria present in the two SLE classifications criteria as an ANA titer detected by IIF on HEP2 cells >1/160 is observed in nearly all SLE patients [between 94 and 100% (33–35)]. The quantity of ANA progressively increases during the 3–5 years preceding SLE clinical expression and diagnosis (2). Consequently, ANA testing represents an essential screening tool because their negativity (titer less than 1/160) makes the diagnosis of SLE extremely unlikely (36). By contrast, their presence, even at higher titer is not SLE-specific as ANA can be produced in a lot of other circumstances such as other CTD, hematologic and hepatic diseases, virus infections, drugs uptake, and in healthy people as previously mentioned. In case of positivity, ANA antigen target(s) must be determined by additional tests with nuclear autoantigens.

#### Clinical Usefulness of ANA Testing

➢ In case of SLE suspicion given clinical symptoms
➢ Importantly, ANAs are useless in SLE follow-up as they remain positive whatever disease activity.

### Antigen Targets of ANA in SLE

#### Anti-Double-Stranded DNA (Anti-dsDNA) AAb

Anti-double-stranded DNA AAbs are present in 43–92% of cases (37–39) with a specificity between 89 and 99% but with variable clinical sensitivities from 8 to 54% (40–46). The methods used for anti-dsDNA AAb detection are numerous, which explains the variability observed in terms of sensitivity. Anti-dsDNA AAbs are quite well identified by nuclear homogeneous IFI pattern (47), but their presence may also be evaluated by quantitative assays such as Farr radioimmunoassay (45), chemiluminescence immunoassay (42, 43), ELISA (46), and fluoro-enzyme immunoassay or by qualitative assays such as immunofluorescence test on *Crithidia luciliae* (CLIFT) (44, 46). For each method, performances will vary according to the manufacturer and the source of the dsDNA (synthetic or purified ds DNA from human or calf origin). Globally, ELISA methods to detect anti-dsDNA antibodies are highly sensitive, but are less specific for the diagnosis of SLE than the immunofluorescence test on CLIFT and the Farr assay as they also detect low-avidity antibodies (48).

Anti-double-stranded DNA AAb positivity is one criteria present in both ACR and SLICC classifications (49). As for the majority of AAb, the specificity of anti-dsDNA AAb in SLE is not of 100% [specificity between 96 and 99% according to the type of test and the published series (40, 41)]. Indeed, they can also be evidenced in the setting of infection, elevation of C reactive protein and in healthy individuals (50). In SLE, the serum level of this AAb is generally correlated with disease activity (51). Moreover, high level of such AAb and their association with anti-Sm antibodies (defined below) are associated with kidney involvement in patients with SLE (52, 53).

##### Clinical Usefulness of Anti-dsDNA AAb Testing

➢ In case of SLE suspicion and ANA > 1/160
➢ In the follow-up of SLE patients when positive at time of diagnosis (the same test in the same laboratory should always be used in this setting).

#### Anti-Nucleosome AAb

The nucleosome is a basic unit of DNA packaging, implicated in the formation of repeating units of chromatin. The anti-nucleosome AAbs are detected in 59.8 and 61.9% of SLE patients’ sera with a sensitivity between 52 and 61% (the highest sensitivity in SLE) and a specificity between 87.5 and 95.7% (54–57). Although, presenting the same nuclear homogenous pattern on Hep2 cells (47), they can be present in the absence of anti-dsDNA AAb and consequently may be helpful for clinicians at diagnosis. In SLE murine models, serum anti-nucleosome AAbs are produced before anti-dsDNA AAb (58). Consequently, the detection of these AAbs may be helpful to establish diagnosis. It is noteworthy that the level of anti-nucleosome AAb (especially IgG3 subtype) is correlated with SLE activity (59). The simultaneous presence of anti-dsDNA, anti-nucleosome, and anti-histone (defined below) AAb has been shown to be associated with severe kidney involvement (54, 60). However, such AAbs have also been detected in patients with mixed connective tissue disease (MCTD) and SSc (56).

##### Clinical Usefulness of Anti-Nucleosome AAb Testing

➢ In case of SLE suspicion and ANA > 1/160 and negative anti-dsDNA AAb
➢ In the follow-up of SLE patients when positive at time of diagnosis (the same test in the same laboratory should always be used in this setting)

#### Anti-Sm AAb

Sm proteins are linked to RNA in the nuclear compartment. Characterized by nuclear coarse speckled pattern on Hep2 cells (47), anti-Sm AAbs are present in 15–55.5% of SLE patients (37–39, 61). These AAbs have a low sensitivity (10–55%) but are very specific for SLE (98–100% according to the test used and to the studied population) and are therefore used in the classification criteria (31, 49, 62).

The main usefulness of anti-Sm AAb detection seems to be in the subset of patients with SLE but without anti-dsDNA AAb, for whom they are present in 14.8% of cases (63). The anti-Sm AAb highlighting in SLE seems to be associated with lupus nephritis (52, 64) and with a poorer prognosis if they are present at the onset of kidney disease (65) and with a higher clinical relevance if they are associated with anti-dsDNA AAb (52, 53). In this line, a recent study showed that the association of a low concentration of complement fraction C3 and signs of complement activity (CH50), together with a high rate of anti-Sm AAb is predictive of lupus nephritis (66). Furthermore, anti-Sm AAbs are mostly expressed in association with anti-RNP (see below) AAb (67). In contrast to anti-dsDNA and anti-nucleosome AAb, anti-Sm AAb level does not correlate with disease activity (68, 69).

##### Clinical Usefulness of Anti-Sm AAb Testing

➢ In case of SLE suspicion and ANA > 1/160 and negative anti-DNA AAb
➢ Not useful in the follow-up of SLE patients
➢ Association with lupus nephritis

#### Anti-Histone AAb

Histones are proteins strongly linked to DNA allowing its compaction, thus forming the nucleosome structure. AAb directed against histone are associated with nuclear homogenous pattern on Hep2 cells (47). In drug-induced SLE such as procainamide, hydralazine, and quinine (70), about 95% of these patients develop anti-histone AAb, whereas these AAbs are only detected in 50–81% of cases of primary SLE (37, 71, 72).

Generally, drug-induced SLE regresses with treatment interruption, and the production of anti-histone AAb decreases alongside the activity of the disease (70, 73, 74).

##### Clinical Usefulness of Anti-Histone AAb Testing

➢ In case of drug-induced SLE
➢ Decreased rate associated with regression of drug-induced SLE

#### Anti-Ro and Anti-La AAb

Anti-Ro (also called anti-SSa) and anti-La (also called anti-SSb) AAbs are often associated with SS but can also occur in SLE with a prevalence between 36 and 64% and between 8 and 33.6% for anti-Ro AAb and anti-La AAb, respectively (37, 38, 61, 75, 76). These antibodies are detected in sera about 3.6 years before SLE diagnosis (2) and commonly give a nuclear fine-speckled pattern on Hep2 cells (47).

In SLE, they are associated with skin (75, 77) and hematologic manifestations such as cytopenia (78). Furthermore, these AAbs are responsible for neonatal lupus by transplacental passage with cardiac, cutaneous, hematologic, hepatobiliary, and neurologic involvement (79). Neonatal lupus occurs in only 2% of female patients with anti-Ro/SSa or anti-La/SSb (80, 81). Maternal autoimmune disease associated with neonatal lupus development is not always SLE, since maternal SLE is responsible for only 15–50% of neonatal lupus cases (79, 82). AAbs directed against the subunit 52 kDa of Ro are associated with a higher risk of congenital heart block (CHB) (41). In more than 90% of neonatal lupus cases, AAb regress within 9 months (82) and only few infants will develop authentic SLE (80, 81). The risk of CHB in these infants may be prevented by maternal treatment with hydroxychloroquine during pregnancy (83, 84). The sensitivity of anti-SSb for SLE is lower than in SS, about 25% and the specificity about 97% (85).

Adult patients with anti-Ro/SSa-positive CTD show a high prevalence of QTc interval prolongation (86), with a direct correlation between anti-Ro52 kDa level and QTc duration (87). In fact, anti-Ro/SSa-positive patients have a particularly high risk of developing complex ventricular arrhythmias (88).

##### Clinical Usefulness of Anti-Ro and Anti-La AAb Testing

➢ In case of skin and hematologic manifestations
➢ Association with CHB

#### Anti-RNP AAb

Anti-RNP AAbs are found in serum from patients with MCTD. In SLE, these AAbs are detected in 23.3–49% of cases (37–39). These AAbs are frequently associated with nuclear coarse speckled pattern on Hep2 cells (47). Clinical sensitivity in SLE is between 8 and 69%, with a specificity between 25 and 82% (62). In contrast with other SLE AAb, anti-RNP AAbs are detected within the year preceding SLE diagnosis (2). However, up to now, correlation with SLE phenotype remains to be clarified.

##### Clinical Usefulness of Anti-RNP AAb Testing

➢ No specific phenotype in SLE
➢ Useless for follow-up

### Non-Antinuclear AAb Frequently Observed in SLE

#### Anti-C1q AAb

Patients with genetic defect in C1q expression have an increased risk to develop a lupus-like disease (89). Anti-C1q AAbs are found in 4–60% of SLE patients, and their prevalence increase with aging (90–93).

High production of anti-C1q AAb is associated with membranoproliferative glomerulonephritis development with 28 and 92% of sensitivity and specificity, respectively (94–97).

These AAbs are detected 2–6 months before lupus nephritis onset (98–100). By contrast, the absence of anti-C1q AAb is associated with less kidney involvement during SLE course (101). These AAbs are also observed in hypocomplementemic urticarial vasculitis associated or not with SLE (also called McDuffie syndrome) (102).

##### Clinical Usefulness of Anti-C1q AAb Testing

In case of lupus nephritis
Also seen in hypocomplementemic urticarial vasculitis

#### Anti-Ribosomal P AAb

Substance P is a neuropeptide that acts as a neurotransmitter and a neuromodulator. Anti-ribosomal P AAb may be detected by very dense fine granular cytoplasmic pattern when testing for ANA on Hep2 cells (47). These AAbs are detected in 12–20% of SLE patients (37, 103, 104) and are associated with disease activity and with neuropsychiatric involvement (105–108). The specificity is between 97 and 100%, and the sensitivity is about 36% (103, 104, 109).

##### Clinical Usefulness of Anti-Ribosomal P AAb Testing

➢ In case of neuropsychiatric lupus
➢ Useless for follow-up

#### Antiphospholipid (APL) AAb

The antiphospholipid syndrome (APS) is observed in 29–46% of SLE patients (110). APS is defined by pregnancy morbidity (mainly fetal losses) and thromboses (arterial and/or venous) in association with the presence of at least one APL AAb [lupus anticoagulant, anticardiolipin (IgM or IgG), and anti-β2 glycoprotein I (IgM or IgG) AAb] on two or more occasions at least 12 weeks apart (111). Some non-thrombotic manifestations such as thrombocytopenia, livedo reticularis, renal microangiopathy, and myelitis can occur in APS but do not belong to classification criteria (112). In SLE, lupus anticoagulant and anticardiolipin are present, respectively, in about 40 and 30% of cases (with or without APS in the same proportion) (113, 114).

Patients having SLE with APS have a threefold higher risk than those without APL to develop a Libman–Sacks endocarditis (115, 116), an increased risk of vascular events (such as thrombosis) and death (113, 114, 117), and a higher risk to develop pulmonary hypertension (118). A global antiphospholipid score is currently developed in SLE to predict thrombotic risk (119).

##### Clinical Usefulness of Anti-APL AAb Testing

➢ In all SLE patients at diagnosis
➢ In all SLE patients regularly during the follow-up and in case of vascular thrombosis, and/or pregnancy morbidity

#### Anti-Aquaporin 4 (AQP4) AAb

Aquaporin 4 is the main water channel in the brain and is also responsible for glutamate and potassium regulation in the blood–brain barrier, synapses, and paranodes adjacent to the nodes of Ranvier. Anti-AQP4 AAb is well known to be specific to neuromyelitis optica (NMO), also called Devic’s syndrome (120).

These AAbs can be detected in SLE and are associated with authentic NMO or atypical NMO (myelitis alone or optic neuritis alone) (121, 122). Anti-AQP4 AAb seem to be strongly associated with anti-Ro/SSa AAb and also, to a lesser extent, anti-dsDNA AAb (122, 123). Nevertheless, these AAbs can also be detected in SLE and persist for years without concurrent clinical or radiological NMO signs (124). These AAbs can be evidenced in the serum, but their detection in the cerebrospinal fluid from patients allows a higher sensitivity and specificity of the test (125).

##### Clinical Usefulness of Anti-AQP4 AAb Testing

➢ Useless for diagnosis
➢ Only if NMO signs

## Sjögren’s Syndrome-Associated AAb

Sjögren’s syndrome is a CTD affecting mainly women, and whose main feature is sicca syndrome. Various organs can be involved in severe forms. Classification criteria include both clinical and immunological parameters (126). Two different forms are observed: primary SS and secondary SS, which is associated with other CTD. The different AAbs observed in patients’ sera and their main features are summarized in Table 3.

**Table 3:** AAb associated with Sjögren’s syndrome.

AAb	Prevalence	Sensitivity	Specificity	Features
Anti-Ro52	33–77.1% (130–134)	42% (130)	100% (130)	CHB++
Anti-Ro60	33–77.1% (130–134)	51% (130)	98% (130)	CHB
Anti-La/SSb	23–47.8% (130–134)	29% (130)	99% (130)	Doubt on pathogenicity
Anti-α-fodrin	98% (141–143)	40% (141–143)	80% (141–143)	–

*CHB, congenital heart block; AAb, autoantibody*.

### ANA in SS

Antinuclear antibody prevalence is estimated between 41.9 and 64% in this disease (127–130). Nevertheless, important discrepancies are observed in the immunological presentation of these patients because the detection of AAb is not mandatory for diagnosis (126). Patients producing high level of ANA with anti-Ro/SSa and/or anti-La/SSb AAb display a more severe disease with various organ involvements.

#### Clinical Usefulness of ANA Testing

➢ Distribution disparity because of classification criteria of SS (immunologic criteria not always required in presence of sicca syndrome and histopathology)
➢ Importantly, ANAs are useless in SS follow-up.

### Targets of ANA in SS

The two main antigens recognized by AAb in SS patients are the Ro/SSa (with two subunits, one of 52 kDa and one of 60 kDa) and the La/SSb antigens. The detection of either anti-Ro/SSa and/or anti-La/SSb AAb constitutes one of the classification criteria but their presence is not mandatory for diagnosis (126). These AAbs are evidenced 4–7 years before SS diagnosis (3, 130).

The sensitivity of anti-Ro52, anti-Ro60, and anti-La is estimated at about 42, 51, and 29%, respectively, whereas the specificity is estimated at about 100, 98, and 99%, respectively (130).

Anti-Ro/SSa AAbs are detected in 33–77.1% of primary SS, whereas anti-La/SSb AAbs are present in 23–47.8% of primary SS (130–134). Anti-Ro/SSa AAb can be observed without anti-La/SSb AAb in patients’ sera, conversely anti-La/SSb alone are rarely evidenced (133). Of note, a recent study reported that the diagnosis of SS was unlikely in patients who had only anti-La/SSb AAb without any anti-Ro/SSa AAb (135).

Concerning disease features, patients displaying both anti-Ro/SSa and anti-La/SSb AAbs are more at risk to develop a non-Hodgkin lymphoma, whereas the absence of those AAbs seems to be associated with a better prognosis (136). In pregnant women, anti-Ro/SSa AAb can induce a high-degree atrioventricular block in fetus in 1–2% of pregnancies (137, 138). This conduction defect seems to be mainly due to anti-52 kDa Ro/SSa AAb (41, 139). Infants of mothers with SS represent 20–30% of neonatal lupus cases (79, 82). Except for cardiac involvement, neonatal lupus signs are completely solved in most of these infants at 9 months of life (82).

In primary SS, anti-Ro/SSa and anti-La/SSb AAbs are associated with earlier disease onset, longer disease duration, greater severity of glandular symptoms, and higher prevalence of extraglandular manifestations (140).

As described previously, adult patients with anti-Ro/SSa-positive CTD show a high prevalence of QTc interval prolongation (86), with a direct correlation between anti-Ro52 kDa level and QTc duration (87). These findings suggest that anti-Ro/SSa-positive patients may have a particularly high risk of developing life-threatening arrhythmias. In fact, anti-Ro/SSa-positive patients have a particularly high risk of developing complex ventricular arrhythmias (88).

#### Clinical Usefulness of Anti-Ro and Anti-La AAb Testing

➢ Association with disease severity (risk of non-Hodgkin lymphoma)
➢ Association with CHB (mostly anti-Ro52) and neonatal lupus by transplacental passage, necessity of screening test and cardiac fetal follow-up in pregnant women at risk
➢ Useless for follow-up

### Non-Antinuclear AAb Observed in SS

#### Anti-Alpha-Fodrin AAb

Alpha-fodrin is an intracellular, actin-binding, organ-specific protein of the cytoskeleton. AAb directed against α-fodrin can be detected in SS in 98% of cases with a sensitivity of about 40% and a specificity of about 80% (141–143). This kind of AAb can also be detected in SLE patients’ sera (141, 144).

These AAbs do not seem to be associated with disease activity or clinical manifestation (145). Anti-α-fodrin AAb could be useful in SS diagnosis when both anti-Ro/SSa and anti-La/SSb were not detected (146).

##### Clinical Usefulness Anti-α-Fodrin AAb Testing

➢ Useful for diagnosis in absence of anti-Ro/SSa and anti-La/SSb AAb
➢ Useless for follow-up

#### Anti-AQP4 AAb

As shown previously, anti-AQP4 AAbs are associated with NMO, also called Devic’s syndrome (120) but can also be detected in SS in association with anti-Ro/SSa AAb in most of cases (122, 123). These AAbs in SS are associated with cranial/peripheral neuropathy, authentic NMO or atypical NMO (myelitis alone or optic neuritis alone) (121, 122). These AAbs can be evidenced both in the serum and in the cerebrospinal fluid (125).

##### Clinical Usefulness of Anti-AQP4 AAb Testing

➢ Useless for diagnosis
➢ Only if NMO signs

## Systemic Sclerosis-Associated AAb

Systemic sclerosis is a CTD characterized by fibrosis, vasculopathy, and autoimmunity. Clinical and immunological expressions of the disease are highly heterogeneous since a large variety of organs can be involved, and various AAbs may be detected in the sera of patients with SSc. Some correlations have been observed between clinical expression and AAb type. In addition, some AAbs are listed in the European League Against Rheumatism (EULAR) classification criteria (147). The association of the different antibodies with the SSc variants is detailed in Table 4.

**Table 4:** AAb associated with systemic sclerosis (SSc).

AAb	Prevalence	Sensitivity	Specificity	Clinical features
Anti-Scl70/DNA topoisomerase I	30.1–41.2% (150, 152, 153)	43% (154)	90% (154)	Diffuse SScPF
Anti-centromere	28.2–36.9% (150, 152, 153)	44% (154)	93% (154)	Limited SScPAH
Anti-RNA polymerase III	3.8–19.4% (152, 153, 163, 164)	38% (154)	94% (154)	Diffuse SScScleroderma renal crisis
Anti-U1-RNP	4.8–4.9% (152, 153)	–	–	Limited SScPAHOverlap with SLE or MCTD
Anti-U3-RNP	1.4–8%16–18.5 in AA (152, 153, 181–183)	12% (154)	97% (154)	Diffuse SScPAH
Anti-Pm/Scl	3.1–13% (150, 152, 173)	12.5% (154, 174)	98% (154, 174)	Limited SScOverlap with myositisPFDigital ulcers
Anti-Ku	1.1–4.6% (150, 152, 176, 177)	–	–	Limited SScOverlap with myositis
Anti-Th/To	0.2–3.4% (152, 153)	–	–	Limited SScPAH
Anti-NOR90	6% (150)	–	–	Limited SScPF

*PAH, pulmonary arterial hypertension; AA, Afro-American population; SLE, systemic lupus erythematosus; MCTD, mixed connective tissue disease; PF, pulmonary fibrosis; AAb, autoantibody*.

### ANA in SSc

Antinuclear antibody prevalence is high in SSc, since about 95% of patients’ sera display such AAb (148–150). Various nuclear proteins can be targeted in SSc. Topoisomerase I, centromeric proteins, and RNA polymerase III represent the three most frequent autoantigens recognized in SSc, but numerous other antigens can be identified. Surprisingly and unexplainedly, the production of two different AAbs by a single patient is exceptional (151).

#### Clinical Usefulness of ANA Testing

➢ In case of SSc suspicion given clinical symptoms, negative ANA in suspicion of SSc makes the diagnosis unlikely.
➢ Importantly, ANAs are useless in SSc follow-up as they remain positive whatever disease activity.

### Targets of ANA in SSc

#### Anti-DNA Topoisomerase I AAb (Anti-Scl70 AAb)

Type I DNA topoisomerases are enzymes that cut one of the two strands of double-stranded-DNA, relax the strand and reanneal the strand. Anti-DNA topoisomerase I AAbs are detected in 30.1–41.2% of SSc sera (150, 152, 153) with a sensitivity estimated about 43% and a specificity about 90% (154). Classically, the associated immunofluorescence pattern on Hep2 cells is speckled and nucleolar (155, 156).

These AAbs are associated with diffuse systemic sclerosis (dSSc) and with a higher risk of pulmonary fibrosis (PF) (157, 158). Two studies reported that anti-Scl70 (a topoisomerase I protein) AAb levels were correlated with disease activity (159) and that negativation of their detection was associated with a better prognosis (160). Nevertheless, these results remain controversial, and their follow-up during disease evolution is not anymore recommended nowadays. Survival rate at 10 years after diagnosis in patients producing those AAbs is estimated at 66% (161).

##### Clinical Usefulness of Anti-Scl70 AAb Testing

➢ Association with diffuse SSc and PF
➢ Not recommended for follow-up nowadays

#### Anti-Centromere AAb

The centromere is a part of the chromosomal structure that links a pair of sister chromatids. Anti-centromere AAbs are detected in 28.2–36.9% of SSc patients (150, 152, 153) with a sensitivity estimated about 44% and a specificity about 93% (154).

These AAbs are associated with limited systemic sclerosis (lSSc) and with a higher risk to develop pulmonary arterial hypertension (PAH) (157, 158, 162). Survival rate at 10 years of patients with anti-centromere AAb, about 93%, is better the one those from patients with anti-Scl70 AAb (161).

##### Clinical Usefulness of Anti-Centromere AAb Testing

➢ Association with limited SSc with a good prognosis
➢ Association with PAH
➢ Useless for follow-up, not correlated with disease activity

#### Anti-RNA Polymerase AAb

RNA polymerase III is used to transcribe DNA into small RNA. Characterized by fine-speckled nucleoplasmic stain with additional occasional bright dots on Hep2 cells (47), anti-RNA polymerase III AAbs are detected in 3.8–19.4% of SSc sera, depending on ethnic group (152, 153, 163, 164) with a sensitivity about 38% and a specificity about 94% (154).

These AAbs are associated with dSSc and with a higher risk of scleroderma renal crisis, gastric antral vascular ectasia (also called watermelon stomach), and cancer (mainly synchronous breast cancer) (157, 165–167). Patients with anti-RNA polymerase III have a higher Rodnan skin score (used for skin fibrosis graduation) than patients with other AAbs and also are more likely to be rapid progressor (167, 168). Survival rate at 10 years in patients producing these AAbs is low, about 30% (161).

Other polymerases can be targeted by self-reactive lymphocytes. Anti-RNA polymerase I AAb may also be produced by SSc patients, mainly in association with anti-RNA polymerase III AAb production. Of note, the detection of isolated anti-RNA polymerase I AAb is not associated with SSc (169). The presence of both anti-RNA polymerase I/III AAb is also associated with cancer and scleroderma renal crisis (170, 171). Furthermore, the concomitant production of anti-RNA polymerase II and III AAb seems to increase the risk of scleroderma renal crisis as compare to the production of anti-RNA polymerase III AAb alone (172).

##### Clinical Usefulness of Anti-RNA Polymerase AAb Testing

➢ Mostly concerning anti-RNA polymerase III AAb in clinical practice
➢ Association with risk of scleroderma renal crisis
➢ Cancer must be search (mostly breast cancer)
➢ Useless for follow-up, not correlated with disease activity

#### Anti-Pm/Scl AAb

Anti-Pm/Scl AAbs are detected in 3.1–13% of SSc patients (150, 152, 173) with a sensitivity about 12.5% and a specificity about 98% (154, 174). Anti-Pm/Scl AAbs are distinguished by homogeneous nucleolar pattern by IFI (47).

These AAbs are associated with lSSc, overlap syndrome with myositis, PF, and digital ulcers (157, 174, 175). By contrast, PAH is less frequent in patients producing those AAbs (174).

##### Clinical Usefulness of Anti-Pm/Scl AAb Testing

➢ Mostly seen in overlap syndrome with myositis
➢ Less likely to be associated with PAH
➢ Useless for follow-up, not correlated with disease activity

#### Anti-Ku AAb

Anti-Ku AAbs are detected in 1.1–4.6% of SSc sera (150, 152, 176, 177), frequently associated with nuclear fine-speckled pattern on Hep2 cells (47). They are associated with lSSc and with a higher risk of myositis and interstitial lung disease (ILD) (150, 157, 177), the absence of digital ulcers and telangiectasia (176).

##### Clinical Usefulness of Anti-Ku AAb Testing

➢ Rarely seen in practice
➢ Useless for follow-up, not correlated with disease activity

#### Anti-Th/To AAb

Anti-Th/To AAb can be detected in 0.2–3.4% of SSc patients (152, 153) with homogeneous nucleolar fluorescence such as anti-Pm/Scl AAb (47). These AAbs are associated with lSSc and a higher risk of PAH (157, 162). A recent long-term follow-up study evidenced that patients with anti-Th/To AAbs are more likely to develop pulmonary hypertension (PAH or pulmonary hypertension secondary to ILD) with a better prognosis and less joint involvement than other SSc patients with other AAbs (178).

##### Clinical Usefulness of Anti-Th/To AAb Testing

➢ Rarely seen in practice
➢ Association with pulmonary hypertension (PAH or pulmonary hypertension secondary to ILD)
➢ Useless for follow-up, not correlated with disease activity

#### Anti-RNP AAb

Anti-U1-RNP AAbs, distinguished by nuclear coarse speckled pattern by IFI (47), are found in 4.8–4.9% of SSc patients (152, 153). They are associated with lSSc and with a higher risk to develop PAH (157). Patients with anti-U1-RNP AAb-associated PAH seems to have a better prognosis than SSc related-PAH associated with other antibodies (179). The presence of this kind of AAb evokes an overlap syndrome with other autoimmune diseases, mostly SLE and MCTD (180).

Anti-U3-RNP AAbs (also called anti-fibrillarin AAb), distinguished by clumpy nucleolar pattern on Hep 2 cells (47), are globally detected in 1.4 and 8% of SSc cases, with important differences between the populations studied (150, 152, 153, 181–183) with a sensitivity about 12% and a specificity about 97% (154). However, these AAbs are more frequently detected in Afro-American people (16–18.5%) (183, 184). Fibrillarin is a component of several ribonucleoproteins including a nucleolar small nuclear ribonucleoprotein. These AAbs are frequently associated with rapidly progressive dSSc (with a Rodnan skin score lower than in other dSSc), muscular involvement, and a higher risk of PAH (182). The presence of anti-fibrillarin AAb in Afro-American population is associated with a higher risk of digital ulcers, pericarditis, and gastrointestinal involvement, but in contrast, with less pulmonary involvement (184).

##### Clinical Usefulness of Anti-RNP AAb Testing

➢ In practice, always ask for both U1 and U3-RNP AAb because of clinical differences
➢ Anti-U1-RNP AAb associated with PAH
➢ Anti-U3-RNP AAb frequent in Afro-American people and associated with diffuse SSc
➢ Useless for follow-up, not correlated with disease activity

#### Anti-Ro/SSa AAb

Anti-Ro/SSa AAbs, also evidenced in SLE and in the SS, are detected in 15–19% of SSc patients, especially AAb directed against the 52 kDa subunit (185). Conversely, anti-SSb AAbs are rarely observed in SSc.

Patients with anti-Ro/SSa AAb show a high prevalence of QTc interval prolongation correlated with anti-Ro52 kDa level and with a higher risk to develop complex ventricular arrhythmias (86–88).

##### Clinical Usefulness of Anti-Ro and Anti-La AAb Testing

➢ Not associated with clinical phenotype
➢ Useless for follow-up, not correlated with disease activity

#### Anti-NOR90 AAb

Nucleolus organizer regions (NORs) are chromosomal regions crucial for the formation of the nucleolus. Anti-NOR90 AAbs are directed against a 90 kDa component of NOR and are found in about 6% of SSc patients (150). These AAbs are associated with punctate nucleolar fluorescence on Hep2 cells (47). Anti-NOR90 AAbs seem to be associated with lSSc and PF (150). These AAbs can also be detected in patients with SLE, SS, and RA (186).

##### Clinical Usefulness of Anti-NOR90 AAb Testing

➢ Rarely seen in practice and not specific to SSc
➢ Useless for follow-up, not correlated with disease activity

#### Anti-Histone AAb

Anti-histone AAbs are evidenced in some SSc sera and seem to be associated with critical internal organ involvement such as cardiac, pulmonary, and renal involvement, and with a decreased survival rate (187, 188).

##### Clinical Usefulness of Anti-Histone AAb Testing

➢ Rarely seen in practice
➢ Useless for follow-up, not correlated with disease activity

### Non-Antinuclear AAb Frequently Observed in SSc

#### Anti-Citrullinated Protein/Peptide AAb (ACPA)

These AAbs are commonly observed in patients with RA but can also be detected in 10% of SSc patients (189). In a recent meta-analysis, the presence of this kind of AAb in the setting of SSc was associated with dSSc, erosive arthritis, and PF (189).

##### Clinical Usefulness of ACPA Testing

➢ Association with erosive arthritis (means overlap syndrome with RA?)
➢ Useless for follow-up, not correlated with disease activity

## Myositis-Associated AAb

Myositis are characterized by a high phenotypic heterogeneity ranging from isolated muscle involvement to various organs manifestations such as ILD, arthritis, or overlap syndrome with other autoimmune diseases. AAbs are currently evidenced in 60–80% of these patients (190, 191). AAbs observed in myositis can be divided in two different groups: myositis-specific AAb (mostly non-ANA) and AAb that can be also observed in other CTD. Four distinct forms of myositis with specific AAbs are currently recognized depending on their clinical and histological features: polymyositis [mainly the antisynthetase syndrome (ASS)], necrotizing myopathy (NM), dermatomyositis (DM), and inclusion body myositis (IBM) (192, 193). In all of these myositis manifestations, only one AAb is detectable in each patient (194). The different myositis-specific AAbs are recapitulated in Table 5.

**Table 5:** AAb associated with myositis [antisynthetase syndrome (ASS), necrotizing myopathy (NM), dermatomyositis (DM), and inclusion body myositis (IBM)].

Kind of myositis	AAb	Prevalence	Clinical and therapeutical features
Anti-synthetase syndrome	Anti-Jo1	70% (194, 197)	Better prognosis More likely associated with myositis than ILD

	Anti-PL7	10% (194, 197)	Poor prognosis

	Anti-PL12	15% (194, 197)	More likely associated with ILD than myositis

	Anti-EJ Anti-OJ Anti-KS Anti-ZO Anti-HA	<2% (194, 197)	–

Necrotizing myopathy	Anti-HMGCR	12–34% (63% with statin history) (202, 205, 206)	Present in statin-associated myopathies Associated with cancer Correlated with disease activity Good response to immunosuppressive treatment (except for statin naïve patients)

	Anti-SRP	18–24% (202, 205)	Correlate with disease activity Associated with ILD Poor response to immunosuppressive treatment

Dermatomyositis	Anti-TIF1-γ	13–38% (212, 213)	Strongly associated with cancer

	Anti-NXP2	17% (212, 216)	Associated with cancer Calcinosis and muscle atrophy in juvenile DM

	Anti-MDA5	10% (40% Asian population) (219, 220)	Associated with severe ILD and skin ulcerations Correlate with disease activityPoor prognosis

	Anti-SAE	7–8% (225, 226)	Severe dysphagia

	Anti-Mi2	18–35% (228, 229)	Good response to immunosuppressive treatments

Inclusion body myositis	Anti-CN1a	30% (231)	Single AAb described in IBM up to now

*ILD, interstitial lung disease; AAb, autoantibody*.

### Anti-Synthetase Syndrome-Associated AAb

Antisynthetase syndrome is characterized clinically by myositis, ILD, arthritis, Raynaud’s phenomenon, mechanic’s hands, fever, and immunologically by the presence of an anti-tRNA synthetase AAb (195). In contrast with other groups of myositis, no correlation with cancer was made in ASS. Amino-acyl-tRNA-synthetases are enzymes that attach the appropriate amino acid onto its tRNA.

The different AAbs describe up to now are the anti-Jo1, anti-PL7, anti-PL12, anti-EJ, anti-OJ, anti-KS, anti-Zo, and anti-Ha AAb. Such AAb, associated with cytoplasmic speckled or fine-speckled fluorescence (47), are detected in about 30% of ASS cases (196). Anti-Jo1 AAb is the most frequently evidenced in about 70% of ASS, followed by anti-PL12 AAb in 15%, anti-PL7 AAb in 10%, whereas other ASS-associated AAbs are observed in less than 2% of the cases (194, 197).

The phenotype and the survival rate depend on the protein targeted by the AAb. Anti-PL7 and anti-PL12 AAbs are mostly associated with ILD and with a worst outcome than anti-Jo1 AAb (198). A long-term follow-up study demonstrated that anti-Jo1 AAb-associated myositis preceded the development of ILD, whereas ILD started before anti-PL7 and PL12 AAb-associated myositis (199). Patients with anti-Jo1 AAb less frequently develop sclerodactyly and ILD but display more frequently myositis than patients producing other types of anti-tRNA synthetase AAb (194). Furthermore, the level of anti-Jo1 AAb seems to be modestly correlated with muscle (in particular serum creatine kinase) and joint activity (200).

#### Clinical Usefulness of ASS AAb Testing

➢ In cases of ASS, mostly anti-Jo1, anti-PL7, and PL12 are detected
➢ Development of myositis first in anti-Jo1 ASS, development of ILD first in anti-PL7 and PL12 ASS
➢ Useless for follow-up, not correlated with disease activity (except for anti-Jo1)

### Necrotizing Myopathy-Associated AAb

Necrotizing myopathy is characterized by subacute proximal limb muscle weakness, strongly elevated creatine kinase levels, muscle fiber necrosis, and regeneration, phenomenon that can be observed on muscle biopsy specimens (201). The two main AAbs in NM are directed against the signal recognition particle (anti-SRP AAb) and the 3-hydroxy-3-methylglutaryl-coenzyme A reductase (anti-HMGCR AAb). These AAbs are present in about 60% of cases (202), and both probably play a pathogenic role in the disease (203, 204).

#### Anti-HMGCR AAb

The 3-hydroxy-3-methylglutaryl-coenzyme A reductase is the rate-limiting enzyme for cholesterol synthesis. The prevalence of anti-HMGCR AAb is of 12–34% (202, 205) and can reach up to 63% in patients with a past history of treatment by statin (206).

Necrotizing myopathy may be associated with cancer, especially when associated with anti-HMGCR AAb (202). Anti-HMGCR antibody serum level seems to be correlated with disease activity and with serum creatine kinase level (207). Generally, NM patients with anti-HMGCR AAb have a good response to immunosuppressive treatments but have a tendency to relapse (208). The presence of anti-HMGCR AAb in statin-naive patients is associated with a lower response to treatment (209).

##### Clinical Usefulness of Anti-HMGCR AAb Testing

➢ Strongly associated with NM with past history of statin treatment
➢ Cancer must be sought for in presence of one of these AAb
➢ Good response to immunosuppressive treatment
➢ Useful for follow-up, correlated with disease activity (and serum creatine kinase level)

#### Anti-SRP AAb

SRP is a complex of six proteins permitting the translocation of nascent proteins to the endoplasmic reticulum. The prevalence of anti-SRP AAb in NM is of 18–24% (202, 205). Like anti-HMGCR, the level of anti-SRP antibody is correlated with disease activity and with serum creatine kinase level (210). Anti-SRP AAbs share also with anti-HMGCR AAb a cytoplasmic dense fine granular pattern by IFI on HEP2 cells (47).

Patients with anti-SRP AAb seem to have more severe muscle weakness and ILD than patients with anti-HMGCR AAb (211). Finally, NM patients with anti-SRP AAb seem to have a reduced response to usual immunosuppressive treatments than other myopathies (208).

##### Clinical Usefulness of Anti-SRP AAb Testing

➢ Association with severe muscle weakness and ILD
➢ Poor response to immunosuppressive treatment
➢ Useful for follow-up, correlated with disease activity (and serum creatine kinase level)

### Dermatomyositis-Associated AAb

Dermatomyositis is an inflammatory disease characterized by proximal muscle weakness and skin involvement. Muscle histology is typical with perifascicular atrophy, vasculopathy, and inflammatory infiltrations. In DM, five AAbs have been described. They are directed against transcription intermediary factor 1 gamma (anti-TIF1-γ AAb), nuclear matrix protein 2 (anti-NXP2 AAb), melanoma differentiation-associated gene 5 (anti-MDA5 AAb), and small ubiquitin-like modifier activating enzyme (anti-SAE AAb), while anti-Mi2 AAbs recognize the nucleosome remodeling histone deacetylase protein complex (NuRD).

#### Anti-TIF1-γ AAb

The TIF1-γ protein (also called TRIM 33 for Tripartite motif-containing 33) is a transcriptional corepressor that acts as a tumor suppressor protein. The anti-TIF1-γ AAb may be detected by nuclear fine-speckled fluorescence on Hep2 cells with a prevalence in DM of 13–38% (47, 212, 213).

The production of AAb directed against this protein is strongly associated with cancer occurrence with a sensitivity of 78%, a specificity of 89%, and positive and negative predictive values of 58 and 95%, respectively (212, 214). These patients are also more frequently diagnosed with dysphagia (215).

##### Clinical Usefulness of Anti-TIF1-γ AAb Testing

➢ Cancer must be sought for in presence of these AAb
➢ Useless for follow-up, not correlated with disease activity

#### Anti-NXP2 AAb

The prevalence of anti-NXP2 in DM is of 17% (212, 216). These AAbs are distinguished by multiple nuclear dots on the nucleoplasm of Hep2 cells by IFI (47). As for anti-TIF1-γ, anti-NXP2 AAb production is associated with a higher risk of cancer development (212). These AAbs are also associated with calcinosis and muscle atrophy, especially in juvenile DM (217, 218).

##### Clinical Usefulness of Anti-NXP2 AAb Testing

➢ Cancer must be sought for in presence of these AAbs
➢ Association with calcinosis, mostly in juvenile DM
➢ Useless for follow-up, not correlated with disease activity

#### Anti-MDA5 AAb

MDA5 is an RIG-I-like receptor functioning as a viral-sensing pattern recognition receptor. The prevalence of anti-MDA5 AAb in DM is of 10% (219) and seems to be higher (about 40%) in Asian population (220).

The presence of anti-MDA5 AAb is associated with a higher risk of developing an ILD (221). Subsequently, patients with this kind of AAb display poorer prognosis, with approximately 50% of death by respiratory failure within the first 6 months following diagnosis (222). Clinically, these patients also present with hand swelling, skin ulceration, panniculitis, and palmar papules (219). Serum level of AAb is correlated with disease activity, and it disappears with its remission (223, 224).

##### Clinical Usefulness of Anti-MDA5 AAb Testing

➢ Poor prognosis with respiratory failure
➢ Mostly, myositis not at the forefront
➢ Useful for follow-up, correlated with disease activity

#### Anti-SAE AAb

SAE is implicated in the nuclear-cytosolic transport and in the transcriptional regulation. The prevalence of anti-SAE AAb in DM is of 7–8% (225, 226) but, in contrast to the anti-MDA5 AAb, the anti-SAE AAbs are less common (about 2%) in the Asian population (227). Clinically, the presence of these AAbs is associated with severe dysphagia (226).

##### Clinical Usefulness of Anti-SAE AAb Testing

➢ Association with severe dysphagia
➢ Useless for follow-up, not correlated with disease activity

#### Anti-Mi2 AAb

Anti-Mi2 AAbs target NuRD, a nuclear proteic complex implicated in multiple transcriptional regulatory processes such as histone demethylation, histone deacetylation, and nucleosome mobilization. They are found in 18–35% of patients with DM (228, 229) and are associated with nuclear fine-speckled fluorescence by IFI on Hep2 cells (47).

Patients with anti-Mi2 AAb seem to have better response to immunosuppressive treatment (229).

##### Clinical Usefulness of Anti-Mi2 AAb Testing

➢ Not associated with a specific clinical phenotype
➢ Useless for follow-up, not correlated with disease activity

### Inclusion Body Myositis-Associated AAb

Inclusion body myositis is a myopathy observed in middle-aged patients that leads to a progressive, asymmetric muscle weakness with swallowing troubles (230). Muscle biopsy evidences vacuolated muscle fibers, inflammatory infiltrates, and intracellular deposits of amyloid protein.

Recently, a novel AAb has been identified (231) in one-third of these IBM patients, which recognizes the cytosolic 5′-nucleotidase 1A (anti-CN1a). Nevertheless, these antibodies are also detected in SLE and in SS patients (232). Its presence or absence does not seem to affect disease prognosis nor evolution (233). This myopathy is poorly responsive to immunosuppressive treatment.

#### Clinical Usefulness of Anti-CN1a AAb Testing

➢ Single AAb described in IBM up to now
➢ Useless for follow-up, not correlated with disease activity

## RheumatoÏd Arthritis-Associated AAb

Rheumatoid arthritis is the most common inflammatory rheumatoid disease with a world prevalence of approximatively 0.5–1% (234). The disease typically affects small and medium-sized joints symmetrically. The primary lesion is synovitis. Systemic involvement is often observed, with respiratory, cardiovascular, and hematopoietic systems being the more damaging lesioned sites.

### Antinuclear AAb in RA

Antinuclear antibody is not the main type of AAb detected in RA but they are present in about 20% of cases (128). The ANA detection has no clinical relevance in RA but is useful for treatments. The highlighting of ANA under infliximab is associated with poorer response to treatment (developing antibody directed against infliximab) and a risk to develop induced lupus (235, 236).

#### Clinical Usefulness of ANA Testing

➢ Useless for diagnosis
➢ Useful in treatment to predict response and complications (induced lupus)

### Non-Antinuclear AAb Frequently Observed in RA

The two main AAb associated with RA (recapitulated in Table 6) are chronologically rheumatoid factor (RF) and ACPA. Other AAbs [anti-CarP (237) and anti-NOR9 0 (186) AAb] are not available in routine practice nowadays. Two main classification criteria are available, based on the presence of both clinical and immunological parameters: the ACR 87 classification (238) and the 2010 classification criteria of the ACR/EULAR (239) collaborative initiative. RF or ACPA measurements between one and three times the upper limit of normal are designated “low”; higher measurements are designated “high.” The high measurement increases the probability of positive diagnosis (238, 239). RA is typically divided into two subtypes designated “seropositive” and “seronegative” disease, with seropositivity being defined as the presence of AAb. The heritability of RA is currently estimated as 40–65% for seropositive RA, but lower (20%) for seronegative disease (240, 241).

**Table 6:** AAb associated with rheumatoid arthritis.

AAb	Prevalence	Sensitivity	Specificity	Features
Rheumatoid factor	50–70% (243)	–	50–95% (245)	Associated with disease activity
ACPA	60–70% (249)	–	95% (243)	Associated with disease activityErosive arthritis

*ACPA, anti-citrullinated peptide AAb; AAb, autoantibody*.

*The term ACPA regroups anti-cyclic citrullinated peptide (anti-CCP) and also anti-non-cyclic citrullinated peptides AAb*.

#### Rheumatoid Factor

Rheumatoid factor is the first well-known RA immunologic marker discover in 1957 (242) that targets the Fc part of human IgG. RFs are present in 50–70% (243) of patients at diagnosis, with little increase throughout disease course (234, 243). There is a correlation between RF titer and radiographic progression (244). The specificity of RF for RA diagnosis depends on clinical context: strong with an articular involvement and low without articular involvement (50–95%) (245).

Rheumatoid factor can also be found in healthy (elderly) individuals and patients with other autoimmune and infectious diseases (245). Despite this lack of specificity, the presence of RF was one of the seven diagnostic criteria for RA put forward by the ACR in 1987 and is also included in the ACR/EULAR 2010 classification criteria for RA.

##### Clinical Usefulness of RF Testing

➢ Useful for diagnosis
➢ Useful in follow-up to predict disease activity

#### Anti-Citrullinated Protein/Peptide AAb

Citrullination is a process by which arginine residues in a given protein are post-translationally modified (“deiminated”) in the presence of high calcium concentrations by an enzyme called PAD (peptidylarginine deiminase) (234, 246). In 1998, two AAbs present in serum samples from patients with RA that had already been described years earlier (antiperinuclear factor and anti-keratin antibodies) were found to share a common specificity for citrullinated filaggrin (247). First, a cyclic citrullinated peptide (CCP) was developed to improve antigen composition and antibody recognition. Then, new assays were developed to detect non-CCPs, and now the term anti-citrullinated protein/peptide AAb (ACPA) has thus replaced anti-cyclic citrullinated peptide (anti-CCP) AAb (248).

Using CCPs as antigens, ACPA are detected in 60–70% of RA patients (249). ACPA appear more specific for RA than RF. The specificity of ACPA is almost of 95% in RA (243). ACPA can also be detected in patients with SSc (189), psoriatic arthritis, SS, SLE, and MCTD (250).

ACPA are linked to erosive form of RA, and the likelihood of radiographic progression after 5 years is significantly greater among RA patients with ACPA (OR = 2.5) (251, 252). Moreover, detection of both RF and ACPA is associated with a more important radiographic progression and a poorer prognostic factor in patients with RA (252). ACPA can be detected in sera several years before clinical onset of arthritis (253). Recently, a new study showed that serological status (ACPA positivity) is a risk factor of serious infusion-related reactions in RA treated by non-TNF-targeted biologics (254).

##### Clinical Usefulness of Anti-ACPA Testing

➢ Useful for diagnosis
➢ Association with erosive arthritis
➢ Useful in follow-up to predict disease activity

## Conclusion

**Figure 1:**
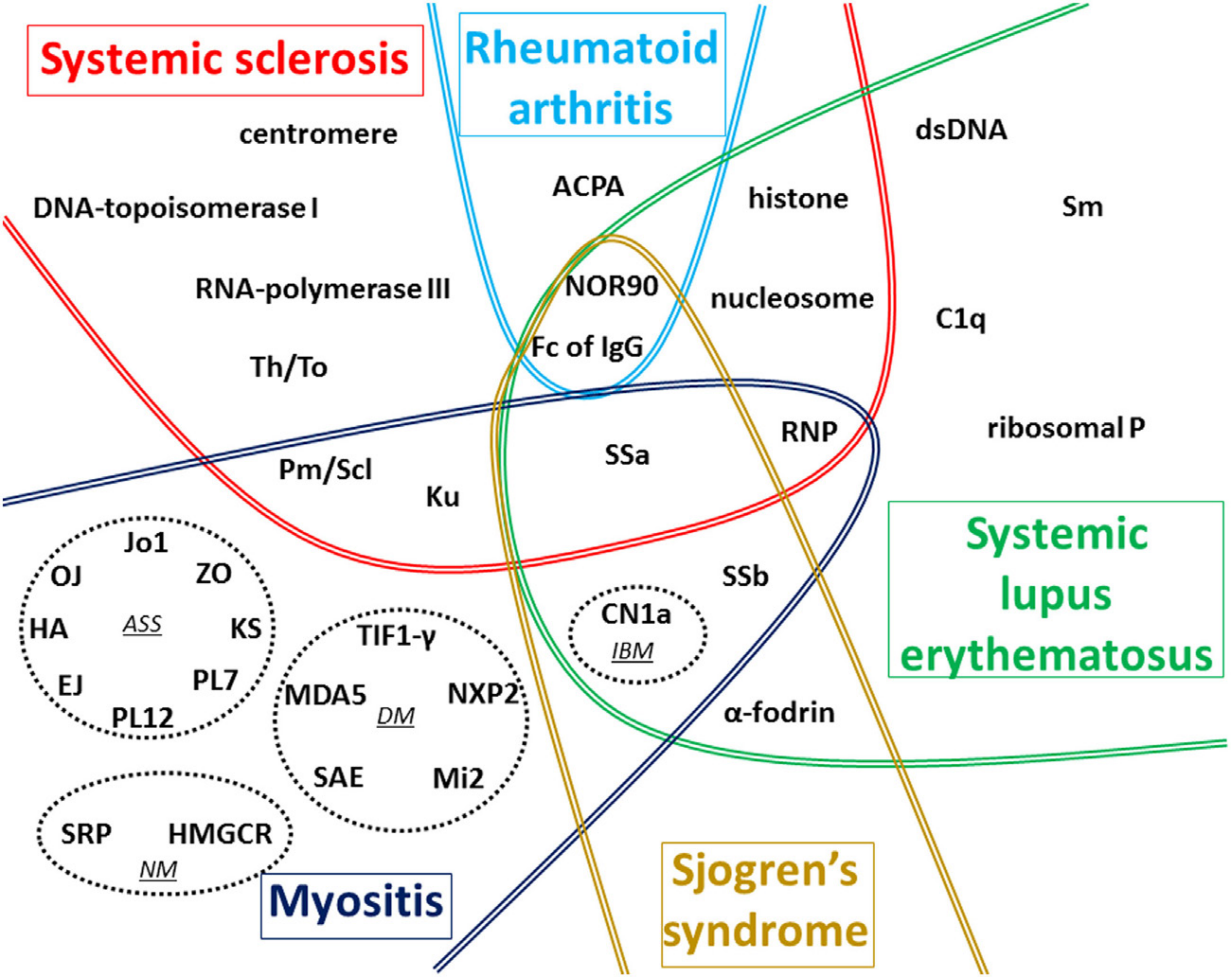
Global vision of autoantigens targeted by autoantibody (AAb) according to the type of connective tissue diseases (CTDs). The main targets of AAb associated with the five CTDs detailed in this review are recapitulated on this figure. In myositis, four distinct forms associated with distinct AAbs are represented in dotted circles: antisynthetase syndrome (ASS), dermatomyositis (DM), necrotizing myopathy (NM), and inclusion body myositis (IBM). In systemic sclerosis (SSc), most AAbs are preferentially associated with one of the two cutaneous forms described: anti-centromere, anti-Th/To, anti-Pm/Scl, anti-Ku, and anti-U1-RNP AAbs are generally associated with limited form of SSc whereas anti-DNA-topoisomerase I, anti-RNA-polymerase III, and anti-U3-RNP AAbs are mostly associated with diffuse cutaneous SSc. The term ACPA regroups anti-cyclic citrullinated peptide and also anti-non-cyclic citrullinated peptides AAb. Fc of IgG corresponds to target of rheumatoid factor. Some AAbs are associated with more than one CTD as shown in the different overlap areas on the figure.

Numerous AAbs can be evidenced in the sera of patients with CTD (Figure 1), and new autoantigens are regularly identified in this field of diseases. In the majority of cases, these AAbs are produced before clinical symptoms, but only a minority of these AAbs has been clearly demonstrated to be involved in the pathogenesis of these diseases. The understanding of the implication in pathogenesis of these AAbs still needs to be investigated, notably using animal models, to be able to find new therapeutic targets.

Evidence of these AAbs can help clinicians for disease diagnosis and is therefore frequently mentioned in international classification criteria. Moreover, since some AAbs are correlated with disease activity and/or specific organ involvement, their detection and in some cases their level follow-up can also be a helpful tool in the long-term management of patients with CTD. The final aim of such investigations would be to personalize medical care according to the CTD and AAb identified.

In conclusion, choice in the type of AAb tested should be carefully evaluated according to clinical context for each patient. Importantly, to properly handle the clinical usefulness of AAb detection, clinician should also be aware of both the advantages and the limits of the methods used to test AAb, to support the clinical evaluation, which remains the essential cornerstone for disease diagnosis and patients’ management.

## Author Contributions

KD and AS designed the review. KD, LB, DG, ST, AR, FA, and AS wrote the manuscript. All the authors critically evaluated the data and approved the final version for publication.

## Conflict of Interest Statement

The authors declare that the research was conducted in the absence of any commercial or financial relationships that could be construed as a potential conflict of interest.

## Abbreviations

AAb, autoantibody; ACPA, anti-citrullinated protein/peptide antibody; ACR, American College of Rheumatology; ANA, antinuclear antibody; anti-dsDNA, anti-double-stranded DNA; AQP4, aquaporin 4; APL, antiphospholipid; APS, antiphospholipid syndrome; ASS, antisynthetase syndrome; CCP, cyclic citrullinated peptide; CHB, congenital heart block; CN1a, cytosolic 5′-nucleotidase 1A; CTD, connective tissue disease; DM, dermatomyositis; dSSc, diffuse systemic sclerosis; EULAR, European League Against Rheumatism; GAPSS, global antiphospholipid score; HMGCR, 3-hydroxy-3-methylglutaryl-coenzyme A reductase; IBM, inclusion body myositis; IIF, indirect immunofluorescence; ILD, interstitial lung disease; lSSc, limited systemic sclerosis; MCTD, mixed connective tissue disease; MDA5, melanoma differentiation-associated gene 5; NM, necrotizing myopathy; NMO, neuromyelitis optica; NOR, nucleolus organizer region; NuRD, nucleosome remodeling histone deacetylase protein complex; NXP2, nuclear matrix protein 2; PAH, pulmonary arterial hypertension; PF, pulmonary fibrosis; RA, rheumatoid arthritis; RF, rheumatoid factor; SAE, small ubiquitin-like modifier activating enzyme; SLE, systemic lupus erythematosus; SLICC, Systemic Lupus International Collaborating Clinics; SRP, signal recognition particle; SS, Sjögren’s syndrome; SSc, systemic sclerosis; TdP, torsade de pointes; TIF1-γ, transcription intermediary factor 1 gamma.
